# Genetic and Functional Characterization of HIV-1 Vpu from HIV-1-Infected North Indian Population

**DOI:** 10.1089/biores.2020.0023

**Published:** 2020-10-13

**Authors:** Jyotsna Singh, Monika Pandey, Vishnampettai G. Ramachandran, Akhil C. Banerjea

**Affiliations:** ^1^ Department of Virology, National Institute of Immunology, New Delhi, India.; ^2^ Department of Transition Medicine, King George's Medical University, Lucknow, India.; ^3^ Department of Microbiology, University College of Medical Sciences, Delhi, India.

**Keywords:** Vpu gene, major histocompatibility complex, HIV-1, Indian population

## Abstract

Acquired immunodeficiency syndrome is a pandemic disease due to increased variability in causative agent in global distribution; it is attributed to various complications in developing the vaccine, namely, error-prone reverse transcriptase, rapid replication, and high recombination rate. Vpu downmodulates CD4 in infected cells, and it targets the newly synthesized CD4 molecules from the endoplasmic reticulum. The aim of this study was to identify the level of genetic changes in the *Vpu* gene from HIV-1-infected North Indian individuals and determine the functional relevance with respect to the CD4 downregulation potential of this protein. Genomic DNA was isolated from peripheral blood mononuclear cells, and the *Vpu* gene was polymerase chain reaction amplified with specific primers followed by cloning, sequencing, and sequence analyses using bioinformatic tools for predicting HIV-1 subtypes, recombination events, conservation of domains, and phosphorylation sites. Among all Vpu variants, three of the variants having serine substitution (serine-52 and serine-56 conversion to isoleucine; S52I and S56I) had lost their functional β-TrcP binding motif. However, the specific determinants for CD4 (V20, W22, S23) and BST-2 (A11, A15, I17, and A19) binding remained highly conserved. The data obtained with Vpu mutants recommend that the serine residue substitutions in cytoplasmic domain distress the CD4 downregulation activity of Vpu. These events are likely to have implications for viral pathogenesis and vaccine formulations.

## Introduction

Acquired immunodeficiency syndrome (AIDS) is a pandemic disease and is complex to study due to its high genetic diversity of causative agent, that is, human immunodeficiency virus (HIV). It is one of the features with attendant consequences in global distribution, success of therapy disease progression, vaccine design, and transmissibility. This extraordinary diversity can be attributed to error-prone reverse transcriptase, rapid replication kinetics, and high recombination rate.^1,2^

HIV is characterized into two types HIV-1 and HIV-2 due to differences in their viral antigens. HIV type-1 is accountable for the AIDS epidemic and consisting of four groups: M (main), O (outlier), N (non-M, non-O), and P.^3,4^ Among these four groups, the M group is successfully established in humans.^5^ The M-group is responsible for a majority of infections worldwide that evolved into a pandemic and diversified into distinct genetic subtypes or clades (A, B, C, D, F, G, H, J, and K),^6^ as well as many circulating recombinant forms (CRFs) and unique recombinant forms. As evidenced by other studies, HIV-1 subtype C prevails worldwide largely in Southern Africa, the Indian subcontinent, and Brazil with an occurrence of 52%,^7^ whereas subtype B prevails in the countries of Western Europe and the United States.^8^ The growing evidence supports subtype-specific difference in pathogenesis and key viral processes, including acquisition of drug resistance.^9^ A majority of studies pertaining to HIV-1 pathogenesis have been carried out on subtype B, whereas little is known about subtype C, which is responsible for the majority of global epidemics (Africa and Asia). In India, subtype C is present throughout the country.^10,11^ Less prevalent HIV-1 subtypes, other than subtype C, have also been reported in different parts of the country. The studies conducted at our laboratory have suggested subtype-specific differences in HIV-1 auxiliary genes (*tat*, *vpr*, *vif, vpu*) with respect to their functions.^12–15^

Recombinant HIV-1 strains are emerging at a high frequency due to cocirculation of multiple HIV-1 subtypes in almost all geographic regions of the world and coinfection of individuals with multiple subtypes. Since *Vpu* is a unique and exclusive feature of the HIV-1 genome, we expected it to show striking genetic variation. Evolution of biological activity of *Vpu* in the HIV-1 genome greatly accelerates virulence. *Vpu* also serves as a classic model to study subtype-specific differences with respect to various biological activities. Earlier in 2013, our laboratory had reported genetic variations in *Vpu* gene from HIV-1-infected North Indian individuals.^15^ Based on similar observations, this study was carried out to see the level of genetic changes in the *Vpu* gene from HIV-1-infected North Indian individuals and determine the functional relevance with respect to the CD4 downregulation potential of this protein.

## Materials and Methods

### Experimental procedures

#### Study participants and sample collection

Blood was drawn from the peripheral vein of the HIV-1-infected individuals residing in the Punjab/Haryana region of North India, selected on a random basis for our studies. Peripheral blood mononuclear cells were extracted from the blood. The samples were obtained from the Immunodeficiency Clinic of the Postgraduate Institute of Medical Research and Education, Chandigarh, India, and ART clinic of Guru Teg Bahadur Hospital, Delhi, India, after obtaining all the required ethical clearances.

#### Ethics statement

The requisite ethical clearance was obtained from Institutional Human Ethics Committee and written consent was taken from all study participants. IRB no.: 324/18.

#### Amplification of the Vpu gene

DNA isolated from the peripheral blood mononuclear cells of infected patients was used to amplify the sequence spanning the *Vpu* gene by polymerase chain reaction (PCR), using gene-specific primers.^15^ PCR was performed with High-fidelity Taq DNA polymerase (Qiagen, Germany) using the following primer pair.

Forward primer Vpu 5′-GGCGAATTCTTATGCAACCTATAATAGTAGCAATAGTAGC-3′

Reverse primer Vpu 5′-GGCGTCGACCTACAGATCATCAATATCCCAAGGAG-3′.

### PCR conditions

The PCR amplification was carried out in a 15 μL of reaction volume. The constituents of reaction mixture were as follows: 500 ng genomic DNA (2.0 μL). Ten times PCR buffer (1.5 μL), 10 mM dNTP mix (0.37 μL), 1 μL of each primer (20 pmol), 0.25 μL Takara Taq polymerase, and 8.88 μL of RNase/DNase-free water. PCR conditions adjusted for the primer set were as follows: initial denaturation at 94°C for 5 min (1 cycle), 30 cycles of denaturation at 94°C for 15 sec, annealing at 63°C for 30 sec, extension at 72°C for 40 sec, and final extension at 72°C for 5 min. The amplified products were checked on 1.5% agarose gel.

### Cloning, sequencing, alignment, and analysis of *Vpu* gene

The *vpu* gene was amplified from genomic DNA samples of HIV-1-infected patients using specific primers engineered to introduce in-frame Myc tag for cloning into pCMV-Myc vector (Clontech, USA). The gel-purified PCR products were cloned into the pGEM^®^-T Easy Vector System (Promega, USA) and also in the expression vector pCMV-Myc (Clontech). The cloning and sequencing were carried out at least twice to rule out the PCR-generated mistakes in the sequence.

Sequencing was performed with T7 and SP6 universal primers using the pGEM-T easy vector. The multiple sequence alignment was done using CLC sequence server (CLC Bio, Denmark) with reference sequences pNL4-3 (AF324493) and 93IN905 (AF067158) prototype as well as HIV-1 strains of all subtypes (www.hiv.lanl.gov) using the Clustal W 1.83 program.^16^ To identify the possible likelihood of our variants, the phylogenetic analysis was constructed with using the neighbor joining method based on the Kimura two-parameter distance matrix implemented in the MEGA 4.0 program.^17–19^

### Cell lines used

Human embryonic kidney 293T (HEK 293T) cells and Tzmbl cells (HIV indicator cells, acquired from NIH, AIDS research reagent) were maintained in Dulbecco's modified Eagle's medium (HizhangMedia) supplemented with 10% fetal bovine serum (FBS; Invitrogen), 100 U/mL penicillin, and 100 μg/mL streptomycin (Invitrogen) at 37°C with 5% CO_2_.

### Immunoblot analysis

Plasmid transfections were performed using Lipofectamine 2000 (Invitrogen) as per the manufacturer's protocol. Relative levels of different proteins were compared by immunoblot analysis. Respective empty vectors were used to normalize transfection. After 48 h of transfection, cells were lysed in RIPA lysis buffer (Cell Signaling Technology), and protein estimation was done using the BCA Protein Estimation Kit (Pierce Biotechnology, Inc.). The primary antibodies used were anti-Myc (Clontech), anti-glyceraldehyde 3-phosphate dehydrogenase (GADPH) (Cell Signaling Technology), and anti-CD4 (Novus Biologicals). The secondary antibodies used were anti-rabbit/mouse-horse radish peroxidase conjugated (Jackson ImmunoResearch). The proteins of interest were detected with EZ western horseradish peroxidase substrate (Biological Industries, Israel). GAPDH was used as a loading control in all cases.

### Cycloheximide chase assay

Cycloheximide (CHX) chase assay was performed to check the kinetic stability of the protein. HEK 293T cells were transfected with 2 μg each of the wild-type and variant Myc-Vpu. After 24 h of transfection, cells were treated with CHX (100 μg/mL), and cell lysates were prepared at indicated time intervals (0, 3, 6, 9, 12, and 14 h) of CHX treatment. Cell lysates were resolved by 12% SDS-PAGE followed by immunoblotting.

### Fluorescent-activated cell sorting assay

Tzmbl cells HIV indicator cells were collected 48 h post-transfection by treating with a cell dissociation buffer. Cells were centrifuged at 1000 rpm at 4°C, the supernatant decanted, and the cells were washed twice with 1 × phosphate-buffered saline (PBS) at 1000 rpm for 10 min. After washing, cells were resuspended in a minimum volume of 1 × PBS and stained with the anti-CD4 antibody on ice for 1 h, followed by analysis on BD FACSVerse.

## Results

### Genetic variation in HIV-1 *Vpu* gene from North Indian HIV-1-infected individuals

**FIG. 1.**
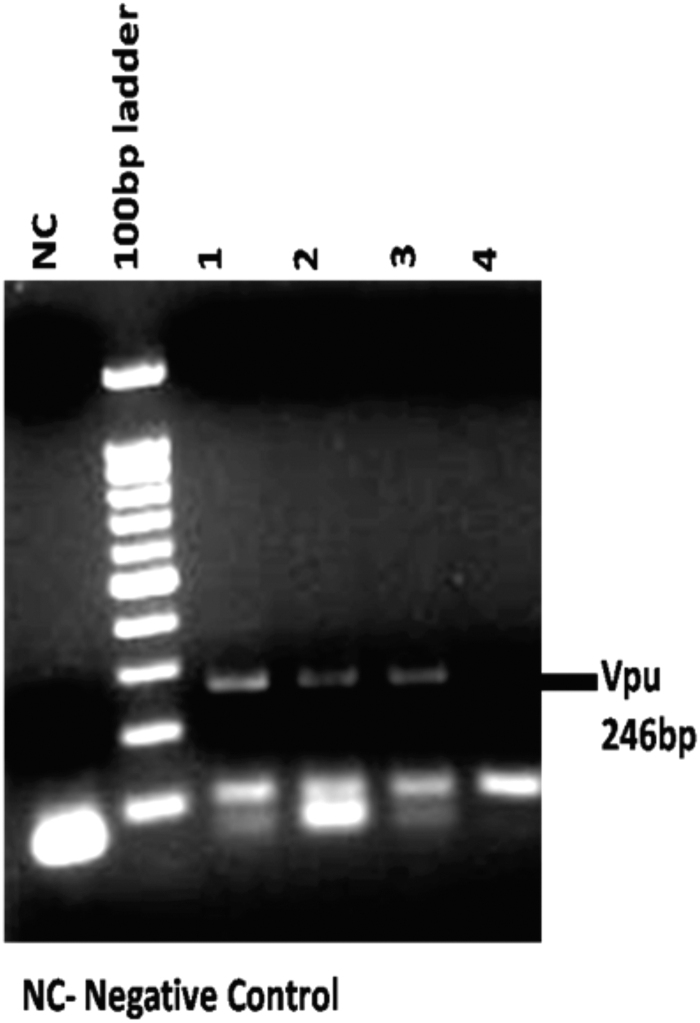
PCR amplification of *Vpu* gene from genomic DNA samples of HIV-1-infected individuals. PCR, polymerase chain reaction.

**FIG. 2.**
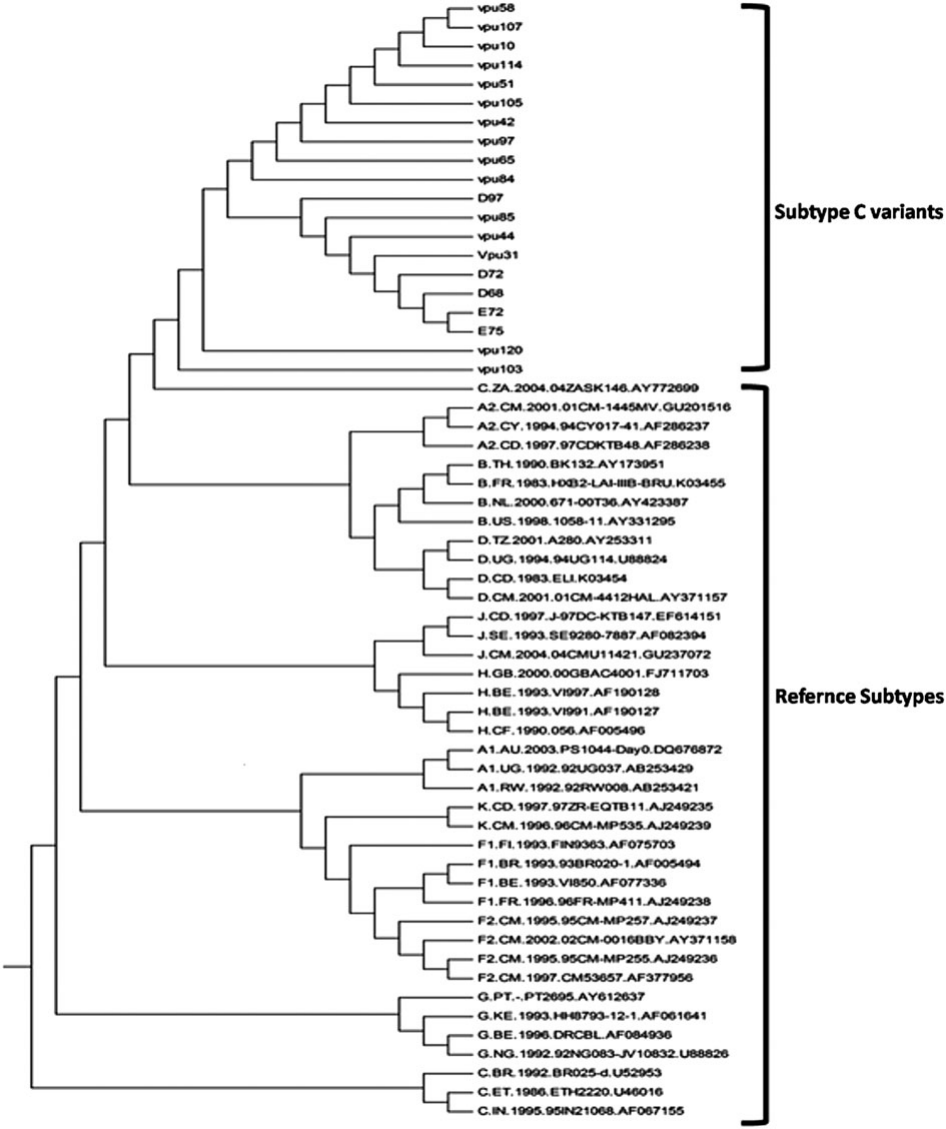
Phylogenetic analysis of Vpu consensus sequences and variant tree. The phylogenetic analysis was carried out using MEGA 4.1 software using the neighbor joining method.

Vpu from subtypes B and C is known to modulate pathogenesis^20^ and also display differential localization^21^ and CD4 degradation activity. Hence, to explore the genetic and functional implication of sequence variations displayed by the Vpu gene from HIV-1-infected patients, Vpu sequences were PCR amplified (Fig. 1), cloned into pGEM-T easy vector, sequenced, and then subjected to genetic- and literature-based characterization. The sequences were subjected to phylogenetic analysis using ClustalW and MEGA version 4 software.^19^ The variants were clustered with prototype subtype C Vpu (Fig. 2).

According to the neutral theory of evolution, the number of synonymous substitutions with synonymous site (dS) is proportional to the rate of nucleotide mutation of a gene. The nonsynonymous/synonymous ratio of rate constants (dN/dS) is indicative of the selection pressures at the protein level. A dN/dS ratio <1 is indicative of purifying selection and amino acid conservation because of functional and structural constraints, and a dN/dS ratio >1 is indicative of diversifying and positive selection where amino acid substitutions confer an advantage.^22^ Interestingly, most of Vpu variants exhibited evidence of positive evolutionary selection as their dN/dS ratio was uniformly greater than 1 (Table 1).

**Table 1.** The Rate of Accumulation of Nonsynonymous and Synonymous Substitution, Calculated by dN/dS Ratio with Consensus B and Consensus C As References

Samples	dN/dS (consensus C)	dN/dS (consensus B)	Predicted subtype	Selection
Vpu D68	0.523	1.841	C	Positive
Vpu D72	0.789	1.841	C	Positive
Vpu E72	0.523	1.841	C	Positive
Vpu E75	0.523	1.841	C	Positive
Vpu D97	0.258	1.687	C	Positive
Vpu 44	1.056	1.783	C	Positive
Vpu 84	0.260	1.808	C	Positive
Vpu 85	0.392	1.844	C	Positive
Vpu 120	0.652	1.838	C	Positive

**FIG. 3.**
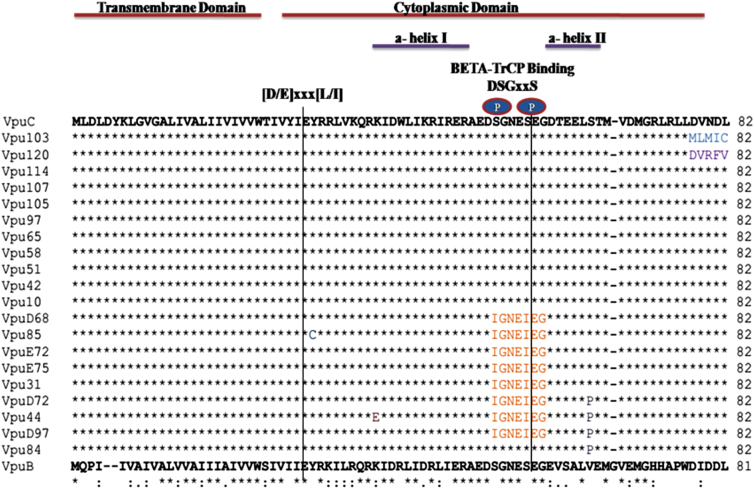
Multiple sequence alignment of unique primary isolates of HIV-1 Vpu. Functional domains are mentioned above the sequences. Dots indicate a match with consensus C subtype and dashes indicate gaps, unique mutations are denoted by different colors.

Multiple sequence alignment of all the Vpu variants is shown with respect to consensus subtypes B and C Vpu (Fig. 3). All the samples resembled prototype subtype C sequence displaying some novel mutations with high allelic frequency (analysis based on unique representative samples). It is noteworthy that the variants displayed variation in the cytoplasmic domain. Interestingly, we noticed for the first time the genetic evidence for positive selection of Vpu variants showing substitution of a serine residue (serine-61 conversion to proline [S61P]) in four of our variants (Vpu D72, Vpu 44, Vpu D97, and Vpu 84).

Among all the eight Vpu variants (Vpu D68, Vpu85, Vpu E72, Vpu E75, Vpu 31, Vpu D72, Vpu 44, and Vpu D97), three of the variants having serine substitution (serine-52 and serine-56 conversion to isoleucine; S52I and S56I) had lost their functional β-TrcP binding motif. As previously reported, the presence of these serine residues in β-TrcP binding motif of Vpu is key to biological function of Vpu and mediates interaction of Vpu with β-TrcP.^23^ Beside these interesting changes, we observed that the specific determinants for CD4 (V20, W22, S23)^24^ and BST-2 (A11, A15, I17 and A19)^25^ binding remained highly conserved (Fig. 3) among all the variants.

### Subcloning and expression of Vpu variants

**FIG. 4.**
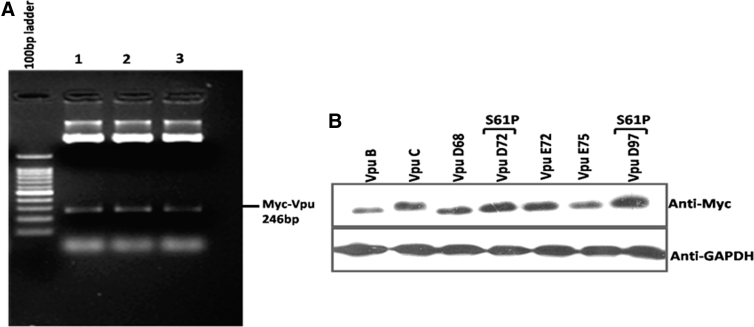
Subcloning and expression of Vpu variants. **(A)** Vpu variants were subcloned into the pCMV-Myc vector and were confirmed by restriction digestion. **(B)** HEK 293T cells were transfected with empty vector/Npu B/Npu C and variants. Immunoblot analysis was done using the anti-Myc antibody. GAPDH was used as a loading control. HEK 293T, human embryonic kidney 293T; GAPDH, glyceraldehyde 3-phosphate dehydrogenase.

Among the Vpu variants, five variants with substitution of serine residue in their β-TrcP binding motif were subcloned with Myc backbone into the pCMV-Myc vector at *Eco*RI and *Sal*I sites. The clones were confirmed by restriction digestion (Fig. 4A). The expression of these variants was checked by transfecting them in HEK 293T cells. After 48 h, cells were harvested and immunoblot analysis was done using the anti-Myc antibody (Fig. 4B).

### Functional characterization of Vpu variants

**FIG. 5.**
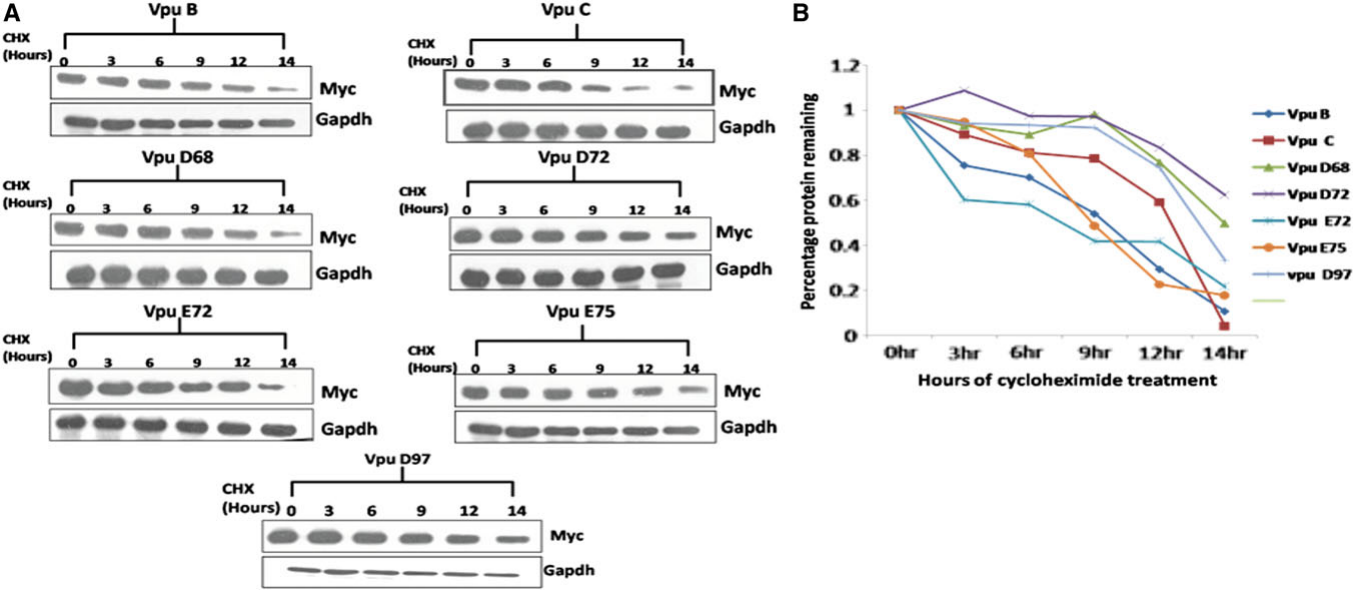
CHX-chase to check kinetic stability of various Vpu variants. **(A)** Cycloheximide chase assay to check kinetic stability of Vpu variants. **(B)** After densitometric quantification, the signals obtained were used to calculate Myc-Vpu wild-type or variant ratios that were plotted against time intervals. CHX, cycloheximide.

It was reported that incorporating mutation at serine residue 61 increases the stability of Vpu protein and also boosts the viral replication rate.^26^ We examined the functional implication of S61P natural mutation found in our variants with respect to intracellular stability and expression. As shown in Figure 4B, different Vpu variants displayed differential migration patterns. Notably, S61P variants (Fig. 4B, lanes 4 and 7) showed higher expression levels than other variants and wild-type Vpu B and C (Fig. 4B). Furthermore, to study the effect of S61P, S52I, and S56I mutations on the kinetic stability of Vpu variants, we performed the CHX chase assay (Fig. 5A). Respective variants and wild-type Vpu were transfected into HEK 293T cells. CHX chase was done at indicated time intervals using 100 μg/mL. Immunoblot analysis was done using the anti-Myc antibody (Fig. 4B). The kinetic stability of all the variants was comparable with the wild-type Vpu B except two variants (Vpu D72 and D97), with substitution of serine residue (S61P) showing no significant reduction in protein levels (Fig. 5B).

### Studying natural Vpu variations with respect to CD4 downregulation potential

**FIG. 6.**
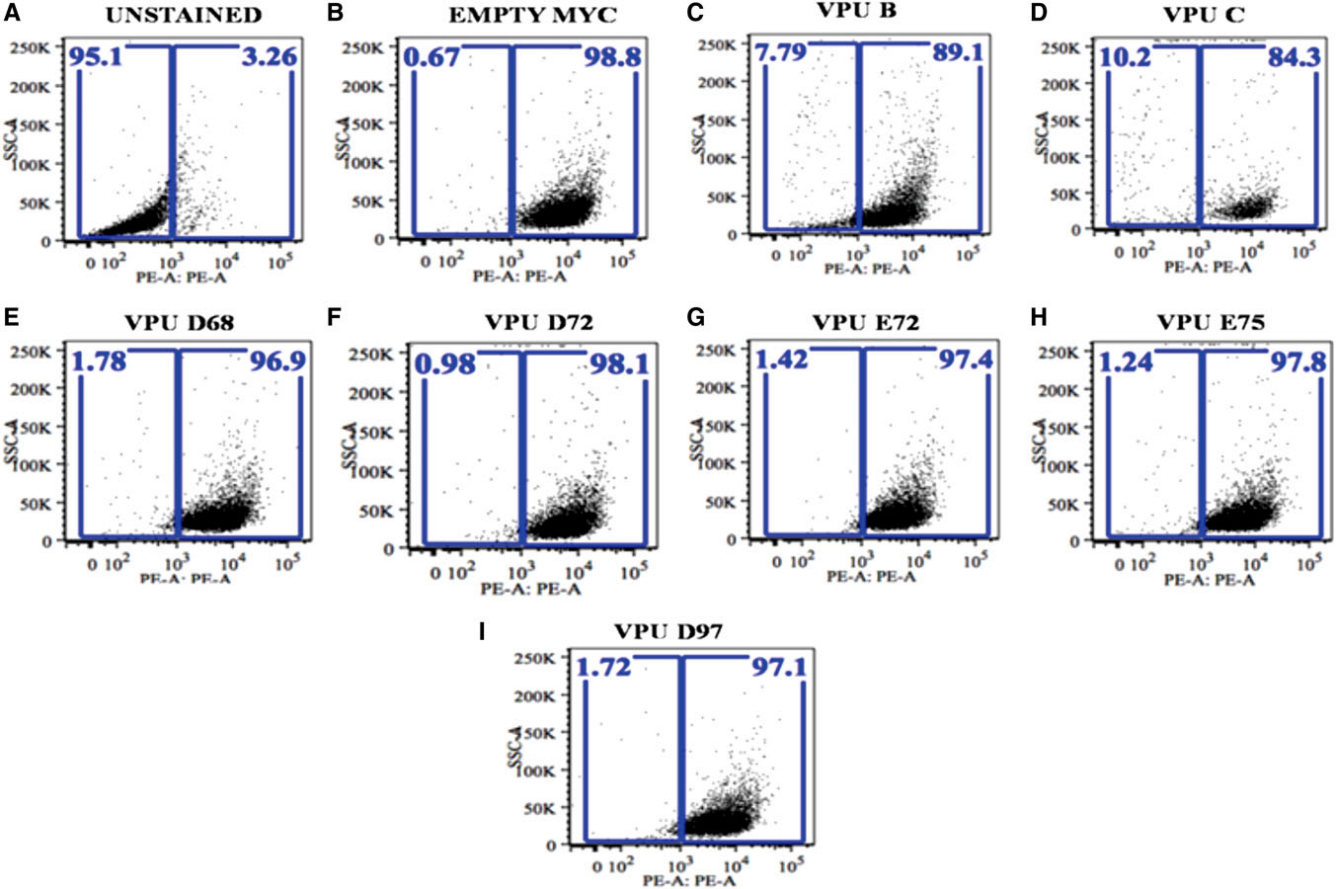
CD4 downregulation potential of Vpu variants. Vpu wild type and mutants were transfected into Tzmbl cells. Forty-eight hours post-transfection, cells were harvested and subjected to flow cytometric analysis.

Since the cytoplasmic domain of Vpu is involved in CD4 degradation,^27^ we further analyzed the functional impact of natural mutation in cytoplasmic domain in our Vpu variants on CD4 downregulation potential. When Tzmbl cells were transfected with wild-type (Fig. 6A–6I) and variant Vpu constructs, Vpu C was more potent in downregulating CD4 (Fig. 6D, 84.3% from 98.8%) in comparison with Vpu B (Fig. 6C, 89.1% from 98.8%). The Vpu mutants showed no downregulation (Fig. 6E, 96.9 from 98.8%; Fig. 6F, 98.1% from 98.8%; Fig. 6G, 97.4% from 98.8%; Fig. 6H, 97.8% from 98.8%; Fig. 6I, 97.1% from 98.8%) and were comparable with empty Myc control (Fig. 6B, 98.8%). The data obtained with Vpu mutants suggest that the serine residue substitutions in cytoplasmic domain affect the CD4 downregulation activity of Vpu.

## Discussion

HIV-1 has remarkable ability to undergo genetic diversity, which helps the virus to adapt itself to varying selection pressures such as antiretroviral therapy. Owing to this high variability of HIV-1, group M has further been divided into distinct subtypes (A to K) and many CRFs, taking into account variations in all genomic regions. Also, genetic changes in alleles derived from infected patients show variations that affect disease progression, viral replication, and drug confrontation.^2^ Hence, studying these genetic changes will help us understand if they confer any survival advantage to the virus and in turn will help design better therapeutics and vaccines. An earlier report in 2013 from our group had already shown differences in the ability of Vpu B and C with respect to differential biological activities.^15^ In the course of genetic analysis of Vpu gene from HIV-1-infected patients from North India, we observed notable variations in the sequence. In general, the variants showed resemblance to subtype C Vpu. Some of our Vpu variants displayed novel mutations with high allelic frequency. Interestingly, we observed that the cytoplasmic domain of some Vpu variants shows substitution of phosphorylated serine residues (S52I, S56I, and S61P). Substitution of serine residue (S61A) in HIV-1 pNL4-3 Vpu was reported earlier to enhance viral replication and stability of Vpu protein.^26^ Therefore, we analyzed whether natural serine residue substitutions in the cytoplasmic regions of Vpu variants displayed a greater kinetic stability. Results of CHX chase assay confirmed a higher kinetic stability associated with S61P mutants. It is noteworthy that when compared with the S61A mutation,^26^ S61P Vpu mutants displayed enhanced intracellular expression and kinetic stability. It is known that the cytoplasmic domain of Vpu is involved in downregulation of CD4 in infected cells. We also observed that CD4 downregulation activity of Vpu variants is comparable with wild-type Vpu B and C. The variants possessing mutant β-Trcp binding motif in their cytoplasmic domain showed no downregulation potential; this is in the agreement with previous reports suggesting the role of β-Trcp binding motif in CD4 downregulation activity.^27^ It is important to develop antiviral approaches against subtype C gene products, since this subtype is responsible for causing more than 50% of the epidemic in the world.^28^

In summary, we were able to show that the North India Vpu C subtype is more widespread. The CD4 downregulation potential of Vpu is contributed by β-Trcp binding motif in the cytoplasmic region, and selection of serine residue substitution (mutant β-Trcp binding motif) in Vpu variants may have important implications in modulating HIV-1 pathogenesis.

## Abbreviations Used

AIDS: acquired immunodeficiency syndrome
CRFs: circulating recombinant forms
CHX: cycloheximide
GAPDH: glyceraldehyde 3-phosphate dehydrogenase
HIV: human immunodeficiency virus
PBS: phosphate-buffered saline
PCR: polymerase chain reaction
